# Understanding the implementation of ‘Making Every Contact Count’ (MECC) delivered by healthcare professionals in a mental health hospital: protocol for a pragmatic formative process evaluation

**DOI:** 10.1080/21642850.2023.2174698

**Published:** 2023-02-05

**Authors:** Angela M. Rodrigues, Emma Kemp, Maria Raisa Jessica Aquino, Rob Wilson, Milica Vasiljevic, Kate McBride, Craig Robson, Mish Loraine, Jill Harland, Catherine Haighton

**Affiliations:** a Department of Psychology, Northumbria University, Newcastle upon Tyne, UK; b Fuse – Centre for Translational Research in Public Health, Newcastle upon Tyne, UK; c Population Health Sciences Institute, Newcastle University, Newcastle Upon Tyne, UK; d Newcastle Business School, Northumbria University, Newcastle-upon-Tyne, UK; e Department of Psychology, Durham University, Durham, UK; f Cumbria, Northumberland, Tyne & Wear NHS Foundation Trust, St Nicholas Hospital, Newcastle upon Tyne, UK; g Northumbria Healthcare NHS Foundation TrustNorth Tyneside General Hospital, North Shields, UK; h Holy Jesus Hospital, Newcastle upon Tyne, UK; i Department of Social Work, Education and Community Wellbeing, Northumbria University, Newcastle Upon Tyne, UK

**Keywords:** Public mental health, healthcare professionals, behaviour change, implementation, health services research

## Abstract

**Background:**

‘Making Every Contact Count’ (MECC) is a public health strategy supporting public-facing workers to use opportunities during routine contacts to enable health behaviour change. A mental health hospital in the North East of England is currently implementing a programme to embed MECC across the hospital supporting weight management (‘A Weight Off Your Mind’). Bespoke MECC training has been developed to improve staff confidence in discussing physical activity, healthy eating, and related behaviour change with service users. This article describes the protocol for a pragmatic formative process evaluation to inform the implementation plan for MECC and facilitate successful implementation of the bespoke MECC training at scale.

**Methods/Design:**

An 18-month, mixed method pragmatic formative process evaluation, including qualitative research, surveys, document review and stakeholder engagement. This project is conducted within a mental health inpatient setting in the North East of England. Programme documents will be reviewed, mapped against MECC national guidelines, Behaviour Change Techniques (BCTs) and intervention functions within the Behaviour Change Wheel. A cross-sectional survey (*n* = 365) and qualitative semi-structured interviews (*n* = 30) will be conducted with healthcare practitioners delivering MECC to assess capability, opportunity and motivation. Data collection and fidelity procedures will be examined, including design, training and delivery dimensions of fidelity. Interviews with service users (*n* = 20) will also be conducted.

**Discussion:**

Anticipated outcomes include developing recommendations to overcome barriers to delivery of and access to MECC, including whether to either support the use of the existing MECC protocol or tailor the MECC training programme. The findings are anticipated to improve fidelity of MECC training within mental health inpatient settings as well as provide evidence for MECC training at a national level. We also expect findings to influence strategic plans, policy, and practice specific to MECC and inform best practice in implementing wider brief intervention programmes.

## Introduction

Serious mental illness (SMI) (e.g. depression, schizophrenia, and bipolar disorders) is the leading cause of ill health and disability worldwide (World Health Organization, 2022). The Marmot review shows that there is a strong socio-economic gradient in SMI, with people from lower socio-economic backgrounds being more susceptible to developing and experiencing SMI (Marmot, 2020). The burden of SMI also seems disproportionately more present in those in poverty or experiencing financial strain when compared to people from higher income groups (Silva et al., 2016). Poor health (physical and mental) increases with increasing socio-economic disadvantage (Whitehead, 2014). Despite the causes of health inequality being relatively similar across the country, the severity of these causes is greater in the North of England (Whitehead, 2014) and the gap has widened over the last four decades (Bambra et al., 2014). The recent Marmot report (2020) highlighted regional differences in life expectancy, the shortest being among people living in deprived areas in the North East of England. Evidence suggests that a combination of material (e.g. income, poor housing), behavioural factors (e.g. smoking as a result of adverse responses) and psychosocial (e.g. social support) contribute to socio-economic health inequalities (Skalická et al., 2009). In a recent prioritisation exercise on how to reduce health inequalities, SMI was consistently rated as an important priority by stakeholders in the North of England (Addison, 2019).

UK data shows that people with SMI have higher rates of cardiovascular disease (e.g. heart attacks and stroke) and have an increased risk of mortality from coronary heart disease, from non-mental health causes, compared to the general population (Skala, 2007; Woodhead et al., 2016). Early mortality may be explained by differences in preventable factors (e.g. unhealthy diet, sedentarism), and access to health care. In the North East of England, the gap in morbidity and mortality between people with SMI and those without is higher than the national average (Collingwood, 2019).

Most physical illness and associated premature mortality experienced by people with SMI is preventable (Campion, 2019). The relationship between SMI and obesity is shown across mental health diagnoses such as depression and schizophrenia (Allison et al., 2009; Avila et al., 2015), which health behaviour change programmes have attempted to address to reduce obesity-related health conditions such as Type 2 diabetes (Allison et al., 2009). Another important consideration is that treatment can contribute to weight gain with almost all antipsychotic medication resulting in weight gain (Bak et al., 2021).

Public mental health (PMH) interventions involve targeted approaches to higher-risk groups to prevent widening of inequalities, such as tertiary prevention strategies for people with SMI to prevent associated impacts of mental illness (Campion et al., 2022). PMH is a national priority and a challenge for healthcare professionals (HCPs) (Walker, 2019). With people with SMI disproportionately impacted by health inequalities, excess weight/sedentary behaviour are important modifiable risk factors affecting health beyond mental health (Osborn, 2007).

Evidence shows that non-pharmacological interventions are effective in reducing body weight in people with SMI (Alvarez-Jimenez et al., 2008; Daumit et al., 2013; Naslund et al., 2017). Nutritional and behavioural weight loss strategies are also associated with improvements in psychological outcomes (Lasikiewicz et al., 2014), with weight loss also improving depressive symptoms and self-esteem (Blaine et al., 2007; Fabricatore et al., 2011). Effective weight management interventions amongst those with SMI include supportive tools such as pedometers or recipe books, ongoing support (e.g. brief telephone calls), and tailored materials and content about the impact of being diagnosed with a mental illness (e.g. low motivation as a result of diagnosis) (Lee et al., 2022).

Extensive literature also shows that brief interventions can be effective in changing several behavioural risk factors (Anderson, 2017; Dewhirst, 2015; Patten, 2016), including diet and physical activity (Dunn, 2001). Implemented in England since 2010, Making Every Contact Count (MECC) is a public health strategy supporting public-facing workers to use opportunities during routine contacts to enable health behaviour change (Public Health England, 2016). HCPs are ideally placed to support and facilitate behaviour change with service users because of their frequent one-to-one contacts with service users. Delivery of opportunistic behaviour change interventions by HCPs is both effective and cost-effective (NICE, 2014; Webb, 2016). Some evidence suggests that HCPs are unsure about their capabilities to facilitate behaviour change with service users unwilling to discuss behaviours perceived as unrelated to the service user’s visit and perceive interventions as burdensome (Keyworth 2018). On the other hand, a place-based evaluation by Harrison et al. (2022) showed that MECC led to positive outcomes including improved staff knowledge and confidence, organisational culture shift and individual behaviour change. In addition, a recent behavioural analysis suggests that intervention developers should incorporate more theoretically relevant components to improve MECC implementation (Haighton et al, 2021).

Bespoke MECC training has been developed to improve staff confidence in having discussions with service users around physical activity and healthy eating in a mental health hospital in the North East of England. The brief advice on physical activity and healthy eating is linked with a regional weight management plan developed at the request of service users to support people with SMI to achieve a healthy weight – A Weight Off Your Mind (AWOYM). Bespoke training has been developed to combine ‘Core MECC’ training with the addition of ‘A Weight Off Your Mind (AWOYM)’. Core MECC is a general training focused on the five core elements of MECC: ‘Physical Activity, Smoking, Alcohol, Healthy Diet & Weight and Mental Health’. AWOYM focuses on Physical Activity and Diet to support service users of inpatient mental health settings achieve a healthy weight which can be achieved through the delivery of MECC conversations. The bespoke MECC training adopts the ‘3As approach’ to brief interventions (‘Ask’; ‘Assist’; ‘Act’) – a shorter and slightly different approach from the widely used 5As (Welzel et al., 2021). It also aims to address the inequalities that people with SMI face, closing the gap between physical and mental health, and reducing inequalities around healthy eating and physical activity. The training implementation started in 2020.

The primary aims of this project are to provide relevant evidence to support the implementation process of MECC in a mental health hospital and to provide recommendations to optimise MECC training. More specifically, this pragmatic formative process evaluation is designed to: (1) explore perceptions of capabilities, opportunities, motivations, and experiences of HCPs delivering MECC; (2) assess service users’ experiences of receiving MECC; (3) assess the theory, techniques and content of MECC as delivered by trained professionals, and report the extent to which it is delivered with fidelity, and is understood by recipients as intended; and (4) identify data collection and management information systems for MECC.

## Method

### Design

The term pragmatic formative process evaluation is used here to describe the process evaluation of a programme currently implemented in routine practice, but lacking systematic development and evaluation (Evans et al., 2015). The proposed pragmatic formative process evaluation includes three work packages using qualitative research, quantitative survey, document review and stakeholder engagement. The Consolidated Framework for Implementation Research (CFIR) (e.g. intervention characteristics) and the Theoretical Domains Framework (TDF) (e.g. Goals, Knowledge), (Atkins et al., 2017; Michie et al., 2005) will be used to identify determinants of implementing MECC (Birken, 2017). Behaviour change techniques (BCTs) will be identified using the Behaviour Change Technique Taxonomy version one (BCTTv1) and the Behaviour Change Wheel (BCW) (Michie et al., 2014). The template for intervention description and replication (TiDieR) (Hoffmann, 2014) will be used to describe key features of MECC training, including mode of delivery, who delivered it, where, and what dose (e.g. duration and frequency). The BCTs in the bespoke training package will be compared to a recent behavioural analysis of nationally available MECC training packages (Haighton et al., 2021).

Ethical approval for this study was granted from Northumbria University Faculty of Health and Life Sciences Ethics Committee (ref 43,190). The protocol was pre-registered and can be found on the Open Science Framework: osf.io/ewktc.

### Public involvement

The initial study proposal was regarded by public contributors (*n* = 5) as important and comprehensive, exploring not only HCPs’ experiences but also implications for users accessing the service. A service user highlighted the importance of assessing the acceptability of providing weight management advice to those with lived experience of SMI (including potential triggers). The research team includes a service user representative (ML) with lived experience of SMI. Public involvement will continue over the course of the project by inviting contributors (*n* = 3; ML plus two other members of the public who are potential users of MECC) to project meetings (*k* = 8) and will support the development of public facing documentation (e.g. lay summary), study protocol (including topic guides), and the interpretation of findings.

### Procedures

#### Quantitative survey

A short quantitative cross-sectional survey will be distributed online to HCPs (*n* = 365) who have responsibility for delivery of MECC (approx. *N* = 7500) at three-time points (T1 = pre-training, T2 = immediately post-training and T3 = 8–10 week follow-up). The sample size estimation for a population size of >7500 included consideration of a 95% confidence interval with a margin error of 5%, so 365 participants will provide sufficient power. The survey will assess the use and delivery of MECC, perceived capabilities, opportunities, and motivations in relation to the delivery of MECC as well as perceived confidence, usefulness, and importance of delivering MECC. We will use a validated questionnaire from a study examining the prevalence of HCPs delivering opportunistic behaviour change interventions (Keyworth et al., 2018). For the survey we will use regression methods to estimate the role of various factors in influencing delivery of MECC in a mental health hospital.

#### Qualitative interviews

##### Healthcare professionals

We will conduct one-to-one, semi-structured telephone interviews with HCPs that have not received MECC training; and those that have received any form of MECC training: ‘train the trainer’ only; bespoke MECC training only or both training components. Training is offered to all staff (not just clinical staff) who (a) have regular conversations with service users, carers or other staff, and (b) who wish to develop skills to be able to support others to make health behaviour changes. For those that have not received training, a multitude of reasons might explain this (e.g. personal choice; job role does not include supporting health behaviour changes; vetted by line manager; time constraints; unaware of training [not regular computer users]). This comparison trained versus non-trained will facilitate understanding of the impact of the bespoke training in delivering MECC in a mental health setting.

We will use a key contact person at the evaluation site, who will help to identify potential HCPs. After obtaining permission, eligible HCPs will be approached individually (via email and/or telephone) by researchers and informed about the study, using an opt-in procedure. They will also be provided with a participant information sheet explaining the purpose of the study.

We estimate 30 interviews (approx. 15 participants in each group). Final sample sizes will be contingent on iterative analysis using information power as a guide (Malterud et al., 2016) and therefore cannot be fully determined in advance of analysis. Topic guides will be iteratively developed in response to feedback from PPI and healthcare professionals’ representatives and early participant interaction. Topic guides will explore the perceived acceptability of the bespoke MECC training particularly in contrast to generic behaviour change, and key enablers and barriers to the implementation of MECC in a mental health setting. We will combine the Consolidated Framework for Implementation Research (CFIR) (Damschroder et al., 2009) (e.g. intervention characteristics) and the Theoretical Domains Framework (TDF) (Atkins et al., 2017) (e.g. Goals, Knowledge) to identify determinants of implementing MECC (Birken, 2017). Interviews will be audio-recorded, transcribed verbatim and analysed using framework analysis (Gale et al., 2013). The analytical framework will reflect the theoretical approach described above.

##### Service users

A qualitative exploration of service users’ experiences of receiving MECC, will be carried out using one-to-one, semi-structured telephone interviews. Service users’ experiences will be explored largely by presenting prompts (e.g. the MECC 3As model) to elicit their experiences and preferences in relation to the process of receiving MECC healthy weight conversations. Interviews will also allow us to explore whether MECC in mental health settings is acceptable to service users and the fidelity of engagement/intervention receipt. We will use a key contact person at the evaluation site, who will help to identify potential service users. After obtaining permission, eligible service users will be approached individually (via email and/or telephone) by researchers and informed about the study, using an opt-in procedure. They will also be provided with a participant information sheet explaining the purpose of the study.

Combining a purposive sampling technique with maximum variance sampling, we will aim to interview up to 20 participants with an SMI with different lived experiences and demographic characteristics. Final sample sizes will be contingent on iterative analysis using information power as a guide (Malterud et al., 2016) and therefore cannot be fully determined in advance of analysis. Interviews will be audio-recorded, transcribed verbatim and analysed using framework analysis (Gale et al., 2013) focussing on:

- Acceptability – the extent to which people receiving the intervention find it appropriate based on anticipated or experienced cognitive and emotional responses to the intervention (Sekhon, 2017).
- Fidelity of engagement (receipt and enactment) – the extent to which the content of MECC is understood by recipients as intended by providers by assessing what they think MECC is trying to do (‘intervention receipt’).

To ensure interrater reliability, three members of the research team (EK, AR, and RA) will independently code the first 10% of participant transcripts line-by-line. These codes will then be mapped to TDF domains (BCTTv1, BCW, TiDieR Framework) through consensus discussion to form the initial coding framework. This coding framework will be applied on a flexible and iterative basis to all remaining transcripts to form a final thematic framework of factors influencing implementation of general and bespoke MECC training delivered at the mental health setting. If codes are linked to more than one area, they will be categorised under the most relevant domain via discussion throughout the analysis process (EK, AR, RA). The final framework will be discussed amongst the research team and again applied to the whole dataset to ensure themes are reflective of participant responses.

#### Intervention fidelity

One of the key dimensions of implementation is intervention fidelity (Moore, 2015), defined as the consistency of what is implemented as specified. Intervention fidelity includes several elements: study design, training, delivery, receipt, and enactment (see below) (Bellg, 2004).

##### Fidelity of design

Document review will be used to identify MECC intervention components/theoretical underpinnings. Protocols, training manuals and materials for the bespoke MECC training will be reviewed. We will code these materials in terms of BCTs, using the BCW (Michie et al., 2014). We will adhere to the provided definitions and guidance on how to assess whether a BCT is present (Michie et al, 2014). We will also use the TiDieR framework (Hoffmann, 2014) to describe key features of the MECC training, including mode of delivery, who delivered it, where, and at what dose (e.g. duration and frequency). We will compare how the BCTs in the bespoke training package compare to a recent behavioural analysis of nationally available MECC training packages (Haighton et al., 2021).

##### Fidelity of training

Observations of MECC training at the mental health setting will be used to assess fidelity of training. We will record and analyse 15 training sessions (according to current implementation plan) from different units across the mental health setting network (including different clinical staff) and code these sessions for BCTs. The number of recordings will be contingent on the available training sessions. These analyses will provide information on the extent to which the bespoke MECC training contains specific BCTs and is theoretically based.

##### Fidelity of delivery

Anonymised consultation notes analysis will provide fidelity of delivery. Consultation notes from different HCPs will be purposively selected to cover variation of professions, units across the mental health setting network, and service user characteristics. Notes will be coded for BCTs and the analysis will assess fidelity of HCPs using specific BCTs. We will examine the extent to which there is a loss of fidelity to the key principles outlined in the bespoke MECC training manual, by comparing numbers of BCTs used and/or missed using standard procedures (Hawkes et al., 2020).

To ensure interrater reliability, three members of the research team (EK, AR, and RA) will independently code a selected 10% of all documents for review. These codes will then be mapped to TDF domains (BCTTv1, BCW, TiDieR Framework) through consensus discussion to form the initial coding framework. This coding framework will be applied on a flexible and iterative basis to all remaining documents to form a final collection of BCTs identified and delivered as part of the general and bespoke MECC training delivered at the hospital. If codes are linked to more than one area, they will be categorised under the most relevant domain via discussion throughout the analysis process (EK, AR, and RA). The final framework will be discussed amongst the research team and again applied to the whole dataset to ensure BCTs are correctly identified.

#### Stakeholder engagement

We will explore the data and management systems used at the mental health setting to collect routine data on MECC delivery. We will involve regional MECC trainers as key stakeholders for this work package with support from the Coordinator of MECC at Scale (CR). We will augment this information through discussions with key stakeholders, PPI representatives and we will consult with relevant people working in Public Health and others with expertise in collection and analyses of healthcare data including systems for assessing data quality where possible.

## Dissemination plan

We will disseminate project outputs through a variety of media, including conference presentations and conventional academic publications, seminars and short accessible reports for stakeholders, and lay summaries and blog entries for the public. We will work closely with stakeholders to maximise the utility of our dissemination. Two of the authors (AR, CH) have previously worked with Public Health England (now Office for Health Improvement and Disparities; OHID) involving a behavioural analysis of barriers and facilitators of delivering Making Every Contact Count (MECC) (Haighton et al., 2021).

We will use established networks to identify MECC stakeholders for dissemination events. We will present findings to relevant stakeholders, including the National MECC Advisory Group, and leading academics in the field. Through presenting these findings, for example at co-hosted workshop events, we aim to initiate stakeholder input, and facilitate wider discussion and problem solving. The aim of this work is to share learnings from the implementation of MECC to date and allow stakeholders to discuss key features in development of a service specification for MECC training packages at a national level.

## Discussion

Public mental health is a national priority and a challenge for HCPs (Walker, 2019). With substantial health inequalities for people with SMI, excess weight/sedentary behaviour are important modifiable risk factors for poor health/reduced life expectancy (Osborn, 2007). MECC is a national public health strategy supporting public-facing workers to use opportunities during routine contacts to enable health behaviour change (Public Health England, 2016). By evaluating a bespoke training package to promote MECC in a mental health setting, study findings will help our understanding of the wider applicability of bespoke MECC training and increase capacity for HCPs delivering MECC across regional and national MECC networks by providing recommendations to optimise bespoke MECC training packages.

The current study addresses the following research needs:

1. To understand barriers and facilitators beyond traditional implementation contexts, such as delivering MECC in a mental health setting (Harrison et al., 2022);
2. To explore the feasibility of delivering MECC with client groups who may be facing an immediate crisis, or a complex or progressive diagnosis such a SMI (Deenik et al., 2019; Harrison et al., 2022);
3. To understand whether tailored support offered through MECC is viewed favourably by services users and HCPs (Harrison et al., 2022; Lee et al., 2022);
4. To contribute to the development of robust monitoring tools that facilitate measurement and attribution of behaviour change outcomes for MECC (Harrison et al., 2022);
5. To provide further evidence on the patient perspective of receiving opportunistic behaviour change interventions (Keyworth et al., 2020) and compare our findings with national and international studies collecting data to identify factors determining delivery of MECC from perspectives of HCPs and service users (Meade et al., 2022).

## Study status

The evaluation described in this protocol is currently at the recruitment stage for the qualitative interviews with healthcare practitioners (HCPs). Document analysis and fidelity checks are in progress.
